# Supplementary material for: Development and validation of a Japanese translation of the K-SF-42
Source: PLoS One. 2022 Sep 16;17(9):e0274217. doi: 10.1371/journal.pone.0274217 (PMC9481016; doi:10.1371/journal.pone.0274217)
Supplement: Attachment — Submitted filename: Response to Reviewers_20220816.doc [file pone.0274217.s001.doc]

Dear Professor Brito-Costa,

We wish to thank you for allowing us to revise and resubmit our manuscript (“*Development and validation of a Japanese translation of the K-SF-42*” [PONE-D-22-10116]). We were pleased to find that both reviewers acknowledged the relevance and potential contribution of our studies. Both reviewers also mentioned several points that they would like to see addressed in a revision. Below, we describe in detail how we have processed the reviewer’s points. By doing so the manuscript has been approved and we hope you may now find it suitable for publication in *PLOS ONE*.

We are looking forward to hearing from you.

Kind regards,

The authors

**RESPONSE TO EDITOR:**

**Comment E.1:** *Please ensure that your manuscript meets PLOS ONE's style requirements, including those for file naming. The PLOS ONE style templates can be found at
https://journals.plos.org/plosone/s/file?id=wjVg/PLOSOne_formatting_sample_main_body.pdf
and https://journals.plos.org/plosone/s/file?id=ba62/PLOSOne_formatting_sample_title_authors_affiliations.pdf*

**Response:** In accordance with the editor’s comment, we have confirmed that our manuscript meets the style requirements of *PLOS ONE*.

**Comment E.2:** *Thank you for stating the following financial disclosure: "This research was supported by JSPS KAKENHI Grant Number JP17K13921."*

*Please state what role the funders took in the study. If the funders had no role, please state: ""The funders had no role in study design, data collection and analysis, decision to publish, or preparation of the manuscript.""*

*If this statement is not correct you must amend it as needed.*

*Please include this amended Role of Funder statement in your cover letter; we will change the online submission form on your behalf.*

**Response:** According to the editor’s comment, we have added the statement that the editor suggested into the cover letter.

**Comment E.3:** *Please review your reference list to ensure that it is complete and correct. If you have cited papers that have been retracted, please include the rationale for doing so in the manuscript text, or remove these references and replace them with relevant current references. Any changes to the reference list should be mentioned in the rebuttal letter that accompanies your revised manuscript. If you need to cite a retracted article, indicate the article’s retracted status in the References list and also include a citation and full reference for the retraction notice.*

**Response:** In accordance with the editor’s comment, we have thoroughly checked the reference list again. Additionally, we have mentioned the new references that we have added into the revised manuscript in this response letter.

Finally, we have added the following text regarding the informed consent (p. 11, lines 248-252 in the revised manuscript):

“The participants read the instructions on the top page of the survey form regarding the informed consent and the voluntary nature of survey participation. Then, only those who agreed to participate in the survey clicked on the link to proceed to the survey content page. Consent to participate in the survey was confirmed by proceeding past the top page of the form.”

Thank you again for the Editor’s comments on our paper. We trust that the revised manuscript is suitable for publication.

**RESPONSE TO REVIEWER 1:**

We wish to express our appreciation to the reviewer for his or her insightful comments, which have helped us to significantly improve the paper.

**Comment R1.0:** *The objective of this study was to demonstrate the internal consistency and construct validity of Japanese translation of the K-SF-42 (J-K-SF-42).
This study has been well conducted. I only have a few comments and suggestions, especially with a view to clearer reporting.*

**Response:** We thank Reviewer 1 for correctly summarizing the purposes and outcomes of our paper as well as for the kind remarks.

**Comment R1.1:** *There are several general classes of reliability estimates. In this study, the authors determined the internal consistency of the J-K-SF-42. I suggest to mention always ‘internal consistency’ instead of ‘reliability’ (see for example lines 20, 449, 495).*

**Response:** According to Reviewer 1’s comment, we have changed “reliability” to “internal consistency” in the whole manuscript.

**Comment R1.2:** *Construct validity should be assessed by testing predefined hypotheses. The authors of this paper did this thoroughly. Notwithstanding this, I challenge them to think about how they can display the confirmation of these hypotheses in a more comprehensive way, for example using a table.*

**Response:** We are uncertain as to the meaning of this comment by Reviewer 1. Is this a suggestion to reiterate the hypotheses and summarize them into one table? If so, we are concerned that the description of the present hypotheses would be redundant in our manuscript.

Correlation coefficients of the subscales of the K-SF-42-J with theoretically-specified variables are already displayed in Tables 7-9, and we believe that these provide adequate tests of predefined hypotheses aimed at supporting construct validity. We respectfully suggest that further tabulations of these same hypothesis tests might neither be necessary nor desirable. To be completely comprehensive in these tabulations, we have calculated the unit-weighted score of the general K-SF-42-J factor and added its descriptive statistics to Table 6. Additionally, we have also added another row to Tables 7-9 that displays the correlations of the unit-weighted score of the general K-SF-42-J factor. These provide tests of convergent validity for the general factor in addition to the subscales of the K-SF-42-J. Regarding this point, we have added the following text to ***“Descriptive statistics”*** section (p. 30, lines 567-569 in the revised manuscript):

“We calculated the unit-weighted score of the general K-SF-42-J factor using the means of the standardized sub-scale scores. Its descriptive statistics are also shown in Table 6.”

Then we have added the following text to ***“Correlation analysis between the K-SF-42-J and related measures”*** section (p. 32, lines 584-585 in the revised manuscript):

“The general K-Factor score was negatively significantly correlated with age. Its average score for women was slightly higher than that for men.”

We have also added the following text to ***“Correlation analysis between the K-SF-42-J and related measures”*** section (p. 32, lines 589-590 in the revised manuscript):

“The general K-Factor score was moderately to highly correlated with these variables with positive direction.”

Additionally, we have added the following text to ***“Correlation analysis between the K-SF-42-J and related measures”*** section (p. 33, lines 605-609 in the revised manuscript):

“The general K-Factor score was positively and significantly correlated with effortful control, habitual exercise, care factor in parental attitudes, social supports from family members, friends, and significant others, altruistic behaviors, and spirituality. The general K-Factor score was negatively significantly correlated with over-protective parental attitudes and avoidant attachment style.”

Moreover, we have changed the following text from (p. 33, lines 641-644 in the revised manuscript):

“All seven subscale scores showed significant positive correlations with the Mini-K score, trait EI, psychological well-being, and cultural and social resources in childhood, except for those of religiosity with trait EI and social resources.”

to

“All seven subscale scores and the general K-Factor score showed significant and positive correlations with the Mini-K score, trait EI, psychological well-being, and cultural and social resources in childhood, except for those of *religiosity* with trait EI and social resources.”

Further, we have added the following text to ***“Discussion”*** section (p. 40, lines 702-706 in the revised manuscript):

“The general K-Factor score was significantly associated with many related variables (see, Tables 7 and 8). Although the K-Factor score was not associated with procrastination, anxious attachment style, and sociosexuality, those observed correlations were generally consistent with the life history theory. This means that the K-SF-42-J is a nice, concise, and well-validated measurement of life history strategies.”

**Comment R1.3:** *Furthermore, I think it would be useful, when evaluating these hypotheses, not only to look at whether there is a correlation between the subscale in question and a particular scale, but also whether this correlation is stronger than the correlation of the other subscales with that scale.*

**Response:** Thank you for this useful suggestion. We completely agree with this comment. However, we are concerned about the inflated number of tests that would be required. If we were to test the differences of correlation coefficients as the reviewer suggests, we would have to do six statistical tests regarding the differences between one particular correlation coefficient (for example, the correlation between *insight* subscale and effortful control) and the other six correlation coefficients for the 14 variables included in the study. This leads to a total of 84 correlation coefficient difference tests. Therefore, the number of tests will increase considerably from the current version. In addition, the correlations among the seven subscales of the K-SF-42-J are moderately high. It is difficult to expect that a variable that should be associated with one subscale is uncorrelated (or weakly correlated) with the other six subscales. For these reasons, we believe that it is appropriate to deal with this suggestion by examining hypotheses on the discriminability of each subscale with another sample.

**Comment R1.4:** *Line 237: the participants received a reward for participation. Is it possible to give some more information on this?*

**Response:** According to Reviewer 1’s comment, we have added the following sentence to the manuscript (p. 11, lines 252-253 in the revised manuscript):

"The participants who completed the questionnaire received approximately $0.5 USD as a reward for participation."

**Comment R1.5:** *Table 1: Since a table must be readable independently, is it possible to list all abbreviations at the bottom of the table? For example K-SF-42-J and Mini-K-J are missing.*

**Response:** According to Reviewer 1’s comment, we have revised this point.

**Comment R1.6:** *Lines 267-268: is the K-SF-42-J a six- or seven-point Likert scale?*

**Response:** We have added the information that the four subscales (insight, planning, and control; romantic partner attachment; general altruism; and religiosity) were rated on a seven-point Likert scale and that the remaining three subscales (mother/father relationship quality; family social contact and support; and friends social contact and support) were rated on a four-point Likert scale.

**Comment R1.7:** *Lines 444-448: maybe some guidelines can be given about how the fit-indices should be interpreted?*

**Response:** We have added the texts regarding the cut-off criteria of the fit-indices into the ***“Statistical analyses”*** section. The revised texts are as follows (p. 20, line 450-p. 22, lines 494 in the revised manuscript):

“First, a confirmatory factor analysis (CFA) was conducted to evaluate the seven-factor structure of the original K-SF-42. We assessed the factor structure (one-factor or seven-factor model) of the K-SF-42-J for all the participants. We evaluated the model fit of the current CFA model using the Bentler-Bonett Comparative fit index (CFI) [64], Tucker-Lewis index (TLI) [65], Root-mean-square error of approximation (RMSEA) [66], and standardized root mean-square residual (SRMR) [67]. Values of CFI and TLI greater than 0.90 are considered satisfactory levels of practical goodness-of-fit; RMSEA values of 0.06 or less are considered indications of good fit, values between 0.06 and 0.08 are considered indications of a mediocre fit, and values greater than 0.08 are considered indications of a poor fit; and SRMR values of 0.08 or less are considered indications of good fit, values between 0.08 and 0.10 are considered indications of a mediocre fit, and values greater than 0.10 are considered indications of a poor fit [68].

Since the total sample included a sample of men and women with a wide range of ages, from 20 to 59 years old, a multi-group CFA was also conducted on the K-SF-42-J, with the sample split by sex and age groups, to assess measurement invariance. The sample was split into two sex groups (men and women) and four age groups (20–29, 30–39, 40–49 and 50–59 years old), and we fitted configural, metric/weak (factor loading equality constraint), scalar/strong (factor loading and intercept equality constraint), and strict invariance models (factor loading, intercept, and residuals equality constraint) using a multi-group CFA approach [69]. Since the chi-square test is highly sensitive to sample size, we preferentially referred to both the absolute and the relative fit indices for model comparison. The absolute fit indices include CFI [64], TLI [65], RMSEA [66], and SRMR [67]. According to Chen, a decrease of less than 0.010 for CFI, an increase of less than 0.015 for RMSEA, or an increase of less than 0.030 for SRMR implies significant deterioration in model fit under testing of measurement invariance [70]. In addition, when testing scalar or strict invariance, that is at the intercept and residual variance levels, an increase of less than 0.010 is recommended for SRMR. The relative fit indices include Akaike's information criterion (AIC) [71] and Bayesian information criterion (BIC) [72]. Both the AIC and BIC provide some advantages; for example, AIC is when dealing with potential model over-fitting, as is often the case with analyses with small sample sizes [73]. Alternatively, BIC is recommended for more conservative model comparisons [74]. Since AIC and BIC have different advantages, it is generally convenient to conduct a model comparison with both fit indices. It is also worth noting that even though the model selection is based on lower AIC and BIC values, additional estimates should be computed, such as the difference (i.e., delta) between the model with the lowest AIC/BIC value and the competing models. Moreover, AIC weights and BIC weights should also be estimated. These metrics indicate the likelihood that at least one of the models under consideration is the best model.

Then we investigated the internal consistency and validity of the K-SF-42-J. The internal consistency of each K-SF-42-J subscale was checked using the Cronbach’s α coefficient. Regarding the validity, we conducted the correlation analyses among related variables to show nomological network of the K-SF-42-J. All analyses were carried out in R 3.5.3, an open source statistical software environment. The *lavaan* package [75] was used for the CFA in structural equation modeling. The package *qpcR* was used for computing AIC weights and BIC weights [76]. The present data and R code were openly available at <https://osf.io/3nmxv/>.”

Then we have added the following three references.

**References**

73.	Hurvich CM, Tsai CL. Regression and time series model selection in small samples. Biometrika. 1989;76(2):297–307.

74.	Kuha J. AIC and BIC: comparisons of assumptions and performance. Sociological Methods & Research. 2004;33(2):188–229.

76.	Ritz C, Spiess AN. qpcR: an R package for sigmoidal model selection in quantitative real-time polymerase chain reaction analysis. Bioinformatics. 2008;24(13):1549-51.

**Comment R1.8:** *Lines 465-469: Why is the AIC and BIC not used to compare the one-factor model with the seven-factor model?*

**Response:** We did not use the relative fit indices in the comparison between the one-factor and the seven-factor models, since these two models’ fit was quite different. However, the model comparisons in the measurement invariance tests were more subtle, which made us to rely on the broad fit indices including the relative indicators (AIC and BIC) and the changes of the absolute fit indices (ΔCFI, ΔTLI, ΔRMSEA, and ΔSRMR). In order to make this point clearer, we have additionally shown the AIC and BIC weights in the model comparisons of measurement invariance tests. Therefore, we have added AIC and BIC weights (in gray) into Table 5 in the revised manuscript.

**Table 5. Model comparison for multi-group measurement invariance disaggregated by sex and by age.**

In addition, we have revised the text in ***“Results”*** section. The revised texts are as follows (p. 23, line 530-p. 24, lines 546 in the revised manuscript):

“Multi-group CFA was conducted on the modified seven-factor model to assess measurement invariance across sexes (Men vs. Women) and age groups (20s, 30s, 40s, vs. 50s). The detailed model fit indices are shown in Table 5. The model comparison for multi-group measurement invariance between men and women revealed that the metric invariance was supported in accordance to the minimum values of AIC and RMSEA, the maximum value of TLI, and a sizeable AIC weight. It is also worth noting that the scalar invariance was supported based on the minimum BIC value and the perfect BIC weight. Based on the cutoff values of change in model fit indices for measurement invariance test [70], the present results supported strict invariance of the K-SF-42-J across sexes (ΔCFIs < 0.010; ΔTLIs < 0.010; ΔRMSEAs < 0.010; and ΔSRMRs ≤ 0.010). The model comparison for multi-group measurement invariance across age groups revealed that the scalar invariance was supported in accordance to the minimum values of AIC and RMSEA, the maximum value of TLI, and the perfect AIC weight. Furthermore, the strict invariance was supported based on the minimum BIC value and the perfect BIC weight. Based on the cutoff values of change in model fit indices for measurement invariance test [70], the present results supported strict invariance of the K-SF-42-J across age groups (ΔCFIs < 0.010; ΔTLIs < 0.010; ΔRMSEAs < 0.010; and ΔSRMRs < 0.010).”

**Comment R1.9:** *Lines 483-487: “According to Chen, … is recommended for SRMR”: this could be moved to the method section.*

**Response:** According to Reviewer 1’s comment, we have moved this text to ***“Statistical analyses”*** section. Please see our response to Reviewer 1’s comment R1.7.

**Comment R1.10:** *Title table 7 and table 8: it must be possible to read the table without reading the text. For example, I suggest to change the title of table 7 into: “Correlation coefficients* *of the K-SF-42-J with the Mini-K-J, trait emotional intelligence, well-being, and cultural and social resources in childhood*”

**Response:** We have changed the titles of Tables 8 and 9 as suggested by Reviewer 1. Please note that the numbering of the tables has been shifted by one due to this revision.

**Comment R1.11.** *Lines 560-562: “In addition, multi-group CFA demonstrated scalar/strong measurement invariance of the K-SF-42-J over the participants’ sexes and age groups.” Explain the implications of this result.*

**Response:** Accordingly, we have revised the following text (p. 37, lines 632-635 in the revised manuscript):

“In addition, multi-group CFA demonstrated scalar/strong measurement invariance of the K-SF-42-J over the participants’ sexes and age groups, which suggests that the translation of the K-SF-42 into Japanese did not limit the measurement validity of the instrument as it generalizes well across sex and age group.”

Finally, the numbering of some references has changed from the original manuscript due to the present revision.

**RESPONSE TO REVIEWER 2:**

We thank the reviewer for his or her fruitful suggestions. We feel the comments have helped us significantly improve the paper.

**Comment R2.0.** *This manuscript describes a Japanese translation of the K-SF-42, a short form of the Arizona Life History Battery (ALHB). Analyses of data from 1440 Japanese online respondents established the instrument's scale reliability and convergent validity.*

**Response:** We thank Reviewer 2 for correctly summarizing the purposes and outcomes of our paper.

**Comment R2.1:** *As a purely methodological project, submitted to an online journal, this work should be published with minor revisions, including improving its English, something that I'm sure one of the native-English-speaking co-authors could do. (For example, there are quite a few missing grammatical articles).*

**Response:** We thank Reviewer 2 for recommending publication with minor revisions, and we have hopefully corrected the remaining errors.

**Comment R2.2:***here've been serious substantive challenges in the recent literature to the ALHB and its offshoots, which I'm sure the authors are aware of, but the review process for this paper isn't the place to do battle over them.*

**Response:** Thank Reviewer 2 for not requiring that we go over these recent critiques yet again, as they have been responded to already on numerous occasions (e.g., Figueredo et al., 2015).

**References**

Figueredo AJ, Cabeza de Baca T, Black CJ, García RA, Fernandes HBF, Wolf PSA, Woodley of Menie MA. Methodologically sound: evaluating the psychometric approach to the assessment of human life history [reply to Copping, Campbell, and Muncer, 2014]. Evol Psychol. 2015;13(2):147470491501300.

**Comment R2.3:** *A seven-factor structure was a better fit to the data than a one-factor structure. What does this result say about the value of the concept of a single, unidimensional K-factor, claimed to represent human psychometric life history strategy?*

**Response:** We have added the following text and table to the revised paper (p. 23, lines 521-529 in the revised manuscript):

“Historically, since the K-Factor was first introduced [8], it has always shown a latent hierarchical structure, with a general factor at the apex of that hierarchy [77,78]. This is not the same thing as a “single, unidimensional K-factor,” but instead what was actually reported on numerous occasions [8,9]. We therefore performed a higher-order factor analysis based on the inter-factor correlation data among the lower-order factors presented in Table 3. As expected, we did find a general, higher-order K-Factor underlying all these lower-order factors that explained 81% of their reliable variance, and featuring the following factor loadings of the higher-order factor on these lower-order indicators. The detailed factor loadings are shown in Table 4.”

Additionally, we have added the following table to show factor loadings of the higher-order K-Factor (p. 28 in the revised manuscript):

**Table 4. Factor loadings of the higher-order K-Factor**

| Subscale | Factor loading |
| --- | --- |
| Insight | 0.50 [0.42, 0.57] |
| Parent | 0.58 [0.51, 0.65] |
| Family | 0.74 [0.67, 0.81] |
| Friends | 0.72 [0.69, 0.76] |
| Romantic | 0.41 [0.36, 0.46] |
| Altruism | 0.65 [0.56, 0.73] |
| Religious | 0.44 [0.36, 0.53] |

*Note*. 95%CIs for correlations are presented in parentheses.

Then we have added the following two references

**References**

77.	Figueredo AJ, Woodley of Menie MA, Jacobs WJ. The evolutionary psychology of the general factor of personality: a hierarchical life history model. In: Buss DM, editior. Handbook of evolutionary psychology. 2nd ed. Hoboken (NJ): Wiley; 2015. p. 943–67.

78.	Figueredo AJ, Peñaherrera-Aguirre M. Life history theory and mating strategies. In: Shackelford TK, editior. The Cambridge handbook of evolutionary perspectives on sexual psychology. Cambridge (UK): Cambridge University Press; 2021. p. 213–41.

**Comment R2.4:** *Some of the instruments chosen to demonstrate convergent validity with K-SF-42-J scales seem to be *too* convergent, so to speak -- so similar to their matched K-SF-42-J scales in item content that high correlations are inevitable. This concern applies to the "Parental Styles of Caregivers," "Social Support," "Altruistic Behavior," and especially the "Adult Attachment" instruments. In view of this, some of the bold-faced correlations in Table 8, although significant, are surprisingly low.*

**Response:** The use of bivariate correlations to indicate convergent validity is never perfect. Convergent validity coefficients are limited by the reliability of measures as well as by confounding by “method variance” (see Campbell & Fiske, 1959; Ferketich et al., 1991; Figueredo et al., 1991).

**References**

Campbell DT, Fiske DW. Convergent and discriminant validation by the multitrait-multimethod matrix. Psychological Bulletin. 1959;56(2):81–105.

Ferketich SL, Figueredo AJ, Knapp TR. Focus on psychometrics. The multitrait-multimethod approach to construct validity. Res Nurs Health. 1991;14(4):315–20.

Figueredo AJ, Ferketich SL, Knapp TR. Focus on psychometrics. More on MTMM: The role of confirmatory factor analysis. Res Nurs Health. 1991;14(5):387–91.

**Comment R2.5:** *Japan has an extraordinarily high frequency of single young adults, many of whom claim to be uninterested in intimate relationships. See for example, https://www.u-tokyo.ac.jp/focus/en/press/z0508_00142.html. This raises the problem of interpreting participants' answers to questions about adult attachment, SOI, and LTMO. What does it mean to describe your romantic attachment style if you've never had a romantic relationship, and don't want one?*

**Response:** We appreciate the information provided by Reviewer 2. The article provided by Reviewer 2 specifically asks about interest in *heterosexual* relationships and there is a rather high probability that not all people within the Japanese population adhere to a heteronormative lifestyle. We should also mention that the Japanese individuals who are reporting not being interested in a romantic relationship also report being lower in income and education. Research has shown that people who have less resource provisioning abilities (education, income, etc.) have less negotiating power on the mating market (Figueredo & McCloskey, 1993), which could potentially discourage one’s participation in the mating market. In the link provided, the authors of the article mention that while disinterested currently, some respondents were still open to the idea of marriage at a later date. In fact, recent research has shown that the proportion of asexual/aromantic people among the Japanese general population is approximately 1% (1.1% for women and 0.3% for men: Hiramori & Kamano, 2020). This supports our notion that some part of the people who don’t “currently” have romantic relationships is actually open to the romantic and/or sexual relationships and that they just suppress or surrender their desires due to various factors like low socio economical status or poor physical health status.

Additionally, romantic attachment style derives partially from parent-child attachment styles that emerge during early development (Bowlby 1969; Olderbak & Figueredo, 2009). While some participants have might not had a direct experience with romantic partner attachment yet, we can assume that they still have an attachment style that derived in development that will manifest in future or possible romantic relationships. Thus, these measures are designed to assess respondent preferences for long term versus short term relationships, not the fact of whether they are currently in one.

**References**

Bowlby J. Attachment and Loss: Volume 1. Attachment. New York: Basic Books; 1969.

Figueredo AJ, McCloskey LA. Sex, money, and paternity: The evolutionary psychology of domestic violence. Ethology and Sociobiology. 1993;14(6):353–79.

Hiramori D, Kamano S. Asking about sexual orientation and gender identity in social surveys in Japan: findings from the Osaka city residents’ survey and related preparatory studies. Journal of Population Problems. 2020;76:443–66

Olderbak S, Figueredo AJ. Predicting romantic relationship satisfaction from life history strategy. Personality and Individual Differences. 2009;46(5–6):604–10.

**Comment R2.6:** *There's apparently a copy-and-paste error on page 16, as the item "Seeing other people exercise motivates me to be more active" appears as an example of both the "Effortful Control" scale and the "Habitual Exercise" scale (where it presumably belongs).*

**Response:** This error has been corrected in accordance with the reviewer's comment.

**Comment R2.7:** *The authors note that additional work should be done to validate this instrument. I would suggest creating Japanese translations of* *other psychometric LH instruments, such as Dunkel's Life History Rating Form, and checking whether the correlations between these instruments in Japanese is similar to their correlations in English.*

**Response:** Accordingly, we have added the following text (p. 39, lines 719-721 in the revised manuscript):

“For example, we might investigate the associations between the K-SF-42-J and other psychometric life history instruments, such as the Life History Rating Form developed by Dunkel et al [83].”

Then we have added the following reference.

**References**

83.	Dunkel CS, Brown NA, Mathes EW, Summerville L, Kesselring SN, Colclasure R. Testing the life history rating form. Evolutionary Behavioral Sciences. 2016;10(3): 202–12.

Finally, the numbering of some references has changed from the original manuscript due to the present revision.
